# Ultrasound-Guided vs. Landmark-Guided Lumbar Puncture for Obese Patients in Emergency Department

**DOI:** 10.3389/fsurg.2022.874143

**Published:** 2022-04-26

**Authors:** Lei Li, Weichen Tao, Xue Cai

**Affiliations:** Department of Emergency Medicine, Shengjing Hospital of China Medical University, Shenyang, China

**Keywords:** emergency department, landmark-guided, lumbar puncture, obese patients, ultrasound-guided

## Abstract

**Objective:**

Emergency patients are in severe and urgent condition. If the patient is obese, the traditional lumbar puncture method is more difficult. This study was to observe the comparison of ultrasound-guided and landmark-guided lumbar puncture for obese patients in the emergency department.

**Methods:**

Sixty patients suspected of intracranial infection, subarachnoid hemorrhage, and intraventricular hemorrhage from January 2018 to June 2020 were selected in the Department of Emergency Medicine, Shengjing Hospital of China Medical University. They were randomly assigned to two groups according to the order of enrollment: Group A (Landmark-guided group, *n* = 30) and Group B (Ultrasound-guided group, *n* = 30). Follow-up assessments were performed to observe lumbar puncture time, the number of bloody CSF, Visual Analog Scale (VAS), the complications, and satisfaction.

**Results:**

Compared with group A, group B had less lumbar puncture time, lower puncture attempts, and a higher first puncture success rate (*P* <0.05). In group B, the number of bloody CSF was less (*P* <0.05), postprocedural low back pain was less (*P* <0.05), intraprocedural sciatic nerve irritation and postprocedural paresthesia were less, but the difference was not statistically significant (*P* > 0.05). Compared with group A, the postprocedural VAS in group B was lower, and the difference was statistically significant (*P* <0.05). The total satisfaction of group A and group B was 60.0 and 86.7%, respectively. The total satisfaction of group B was higher than that of group A (*P* <0.05).

**Discussion:**

Ultrasound-guided lumbar puncture can be used for obese patients with difficulty in the lumbar puncture. It is worthy of clinical application and promotion.

## Introduction

With the development of the social economy and people's living standards, the intake of excessive high–calorie and high-fat foods leads to excessive fat accumulation in the body (1). Therefore, there are more and more obese people. It is reported that currently, nearly one-third of the world's population is overweight (2), and there is an increasing trend year by year (3). Lumbar puncture is a common clinical practice. It is commonly used to collect cerebrospinal fluid (CSF) for performing routine and biochemical testing of CSF, cell smear, bacterial and fungal examinations. It can measure the pressure of the CSF and understand whether the subarachnoid space is obstructed (4). The success of traditional lumbar puncture mainly depends on the accurate positioning of the puncture point. The accurate location of the puncture point was mainly based on the anatomical landmarks, through the Tuffier's line (the transverse line connecting the superior aspects of the iliac crests) and touching the interspace of the spinous process. However, the actual level of Tuffier's line may vary from the L4 body to the L5 body, the line is insufficient to use for assessing spinal segmental level (5). Emergency patients are in a severe and urgent condition, and it is difficult to coordinate with the proper position. If the patient is obese, the subcutaneous fat layer is thickened, the ligament and bony landmarks are not displayed clearly, the traditional lumbar puncture method is more difficult, greatly increasing the possibility of failure.

There are many reports on the technical improvement of lumbar puncture difficulty (6). However, for patients with emergencies, serious illnesses, mobility impairments, and obesity, there are few reports on the solutions to lumbar puncture difficulties. Therefore, this article applies ultrasound-guided to obese patients in the emergency department to comprehensively evaluate the success rate, the incidence of side injuries, and postoperative complications of lumbar puncture.

## Methods

### Patients

**Figure 1:**
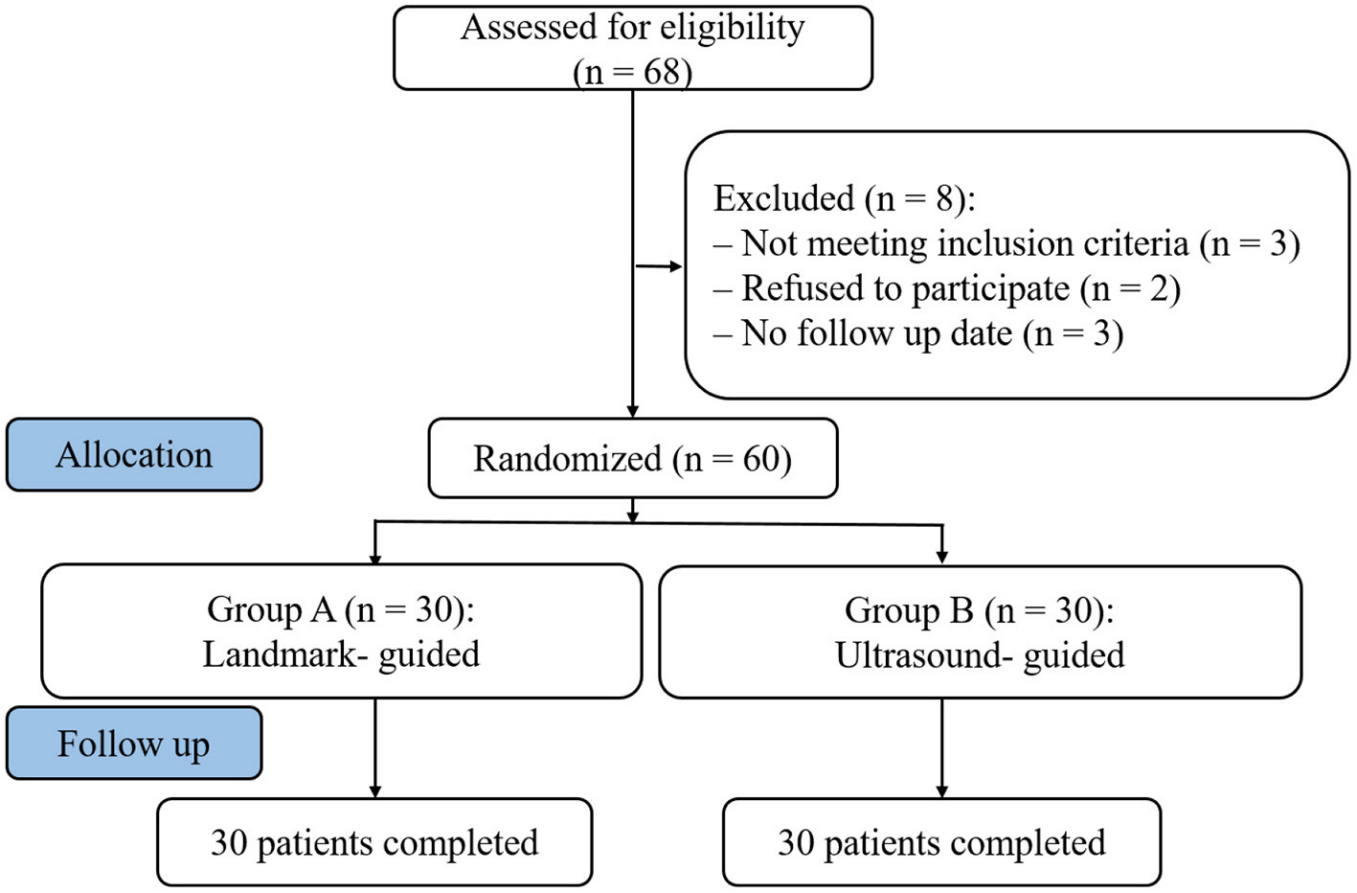
Study flowchart. Of 68 initial patients, 60 patients were randomly assigned and underwent lumbar punctures.

Sixty patients suspected of intracranial infection, subarachnoid hemorrhage, and intraventricular hemorrhage from January 2018 to June 2020 were selected in the Department of emergency, Shengjing Hospital of China Medical University (Figure 1). Patients needed lumbar puncture to confirm the cause or diagnosis, and were randomly assigned to two groups according to the order of enrollment: Group A (Landmark-guided group, *n* = 30) and Group B (Ultrasound-guided group, *n* = 30).

Inclusion criteria: (1) meet the obesity diagnostic criteria. Obesity diagnosis refers to the Chinese WGOC definition (7, 8), Body mass index (BMI) ≥28 kg/m^2^; (2) age >18 years; and (3) stable vital signs and consciousness.

Exclusion criteria: (1) puncture regional infection; (2) Contraindications for lumbar puncture; (3) Combined spinal deformity; (4) mental illness, mental disorders, or disturbance of consciousness such that patient could not cooperate; (5) severe liver, kidney, heart or lung disease; and (6) abnormal coagulation function.

The doctors performing lumbar punctures were all attending physicians with more than 5 years of clinical experience. The study was approved by the Ethics Committee of Shengjing Hospital, China Medical University. All patients were informed of the risks and complications before procedure.

### Surgical Procedure

The patients were in the left lateral position, flexed hips and knees, bent head toward the chest, and backs were aligned near the bed. Group A: Patients performed lumbar puncture using traditional surface landmark–guided, the interspinous space was identified by palpation. Marked the surface according to the axial plane of the iliac crests and perform routine skin disinfection. Group B: Patients performed lumbar puncture using ultrasound-guided. The convex array ultrasonic probe (TOSHIBA Aplio 400 6C1) was used to scan the lumbosacral region in the longitudinal section, and the L5-S1 interspinous space was firstly determined. Gradually move the probe to the head side, perform a cross-sectional scan at the target interspinous space, clarify the epidural and other structures, and determine the most clearly displayed space (L3-4 or L4-5). The left side of the space 1.0–1.5 cm away from the midline is used as the puncture point and marked.

After routine skin disinfection, the puncture point was infiltrated and anesthetized with 1% lidocaine. The physicians fixed the skin, held the needle (20 G lumbar puncture needle), and slowly inserted it in the direction of the head from the patient's L3-4 or L4-5 interspinous space. After breaking through the ligamentum flavum and dura mater, the resistance disappeared and the CSF outflow, proving that the puncture was successful.

### Observations and Follow-Up

Preprocedural data, including gender, age, BMI, interspace level, and preprocedural VAS were recorded. Follow-up assessments were performed before and after the procedure, respectively.

(1) Lumbar puncture time (time from a puncture to CSF outflow), first puncture success rate, puncture attempts, and total puncture success rate;
(2) The number of bloody CSF (CSF red blood cells ≥400/μl due to repeated puncture), except for subarachnoid hemorrhage;
(3) Pain score in puncture: visual analog scale (VAS) was used to assess the degree of pain, painless (0 points) to severe pain (10 points);
The incidence of intraprocedural sciatic nerve irritation, postprocedural low back pain, paresthesia, and other complications;
(4) The evaluation of the degree of satisfaction with the puncture method was divided into three levels: very satisfied, satisfied, and dissatisfied.

### Statistical Analysis

The data were analyzed and processed by SPSS18.0 analysis software. The Kolmogorov-Smirnov test was used to assess the normality of measurement data. The variables with normal distribution were expressed as mean ± standard deviation (x ± SD). The values were compared using one-way analysis of variance, and LSD was used for pairwise comparison. The variables that did not conform to the normal distribution were expressed as the median (interquartile range). The values were compared using the Kruskal-Wallis test. Counting data were analyzed using Chi-square or Fisher's exact test. *P*-values <0.05 were considered statistically significant.

## Results

### Patients Characteristics

The preprocedural patient characteristics in group A and group B were compared. There were no significant differences in gender, age, BMI, interspace level, and preprocedural VAS (*P* > 0.05) (Table 1).

**Table 1:** Preprocedural patients' characteristics in A and B group.

**Parameters**	**Group**		***P***
	**A**	**B**	
Patients (*n*)	30	30	-
Gender (F/M, %)	13 (43.3)/17 (56.7)	11 (36.7)/19 (63.3)	0.792
Age (year, range)	59.87 ± 7.16 (45–74)	59.83± 7.21 (46–75)	0.986
BMI	29.74 ±1.44	29.84 ±1.47	0.793
**Interspace level**			
L3-4	10	9	-
L4-5	20	21	-
Preprocedural VAS	4.03 ± 1.10	4.07 ±0.98	0.902
Viral meningitis	22	21	-
Suppurative meningitis	6	8	-
Tuberculous meningitis	2	1	-

*Data are presented as numbers (%) of patients or mean ± SD*.

### Intraprocedural Conditions

**Figure 2:**
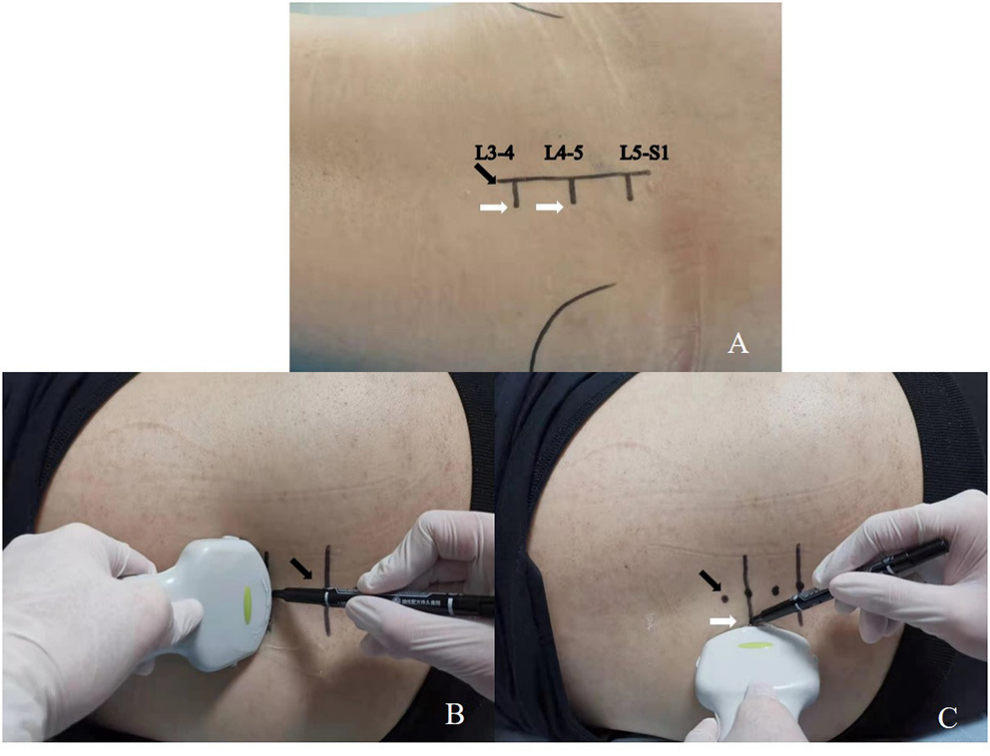
Skin marking before the procedure. **(A)** Mark the skin in the landmark-guided group, midline (black arrow) and possible puncture point (white arrow). **(B)** Mark the skin in the ultrasound-guided group, midline (black arrow). **(C)** Mark the skin in the ultrasound-guided group, puncture point (white arrow).

**Figure 3:**
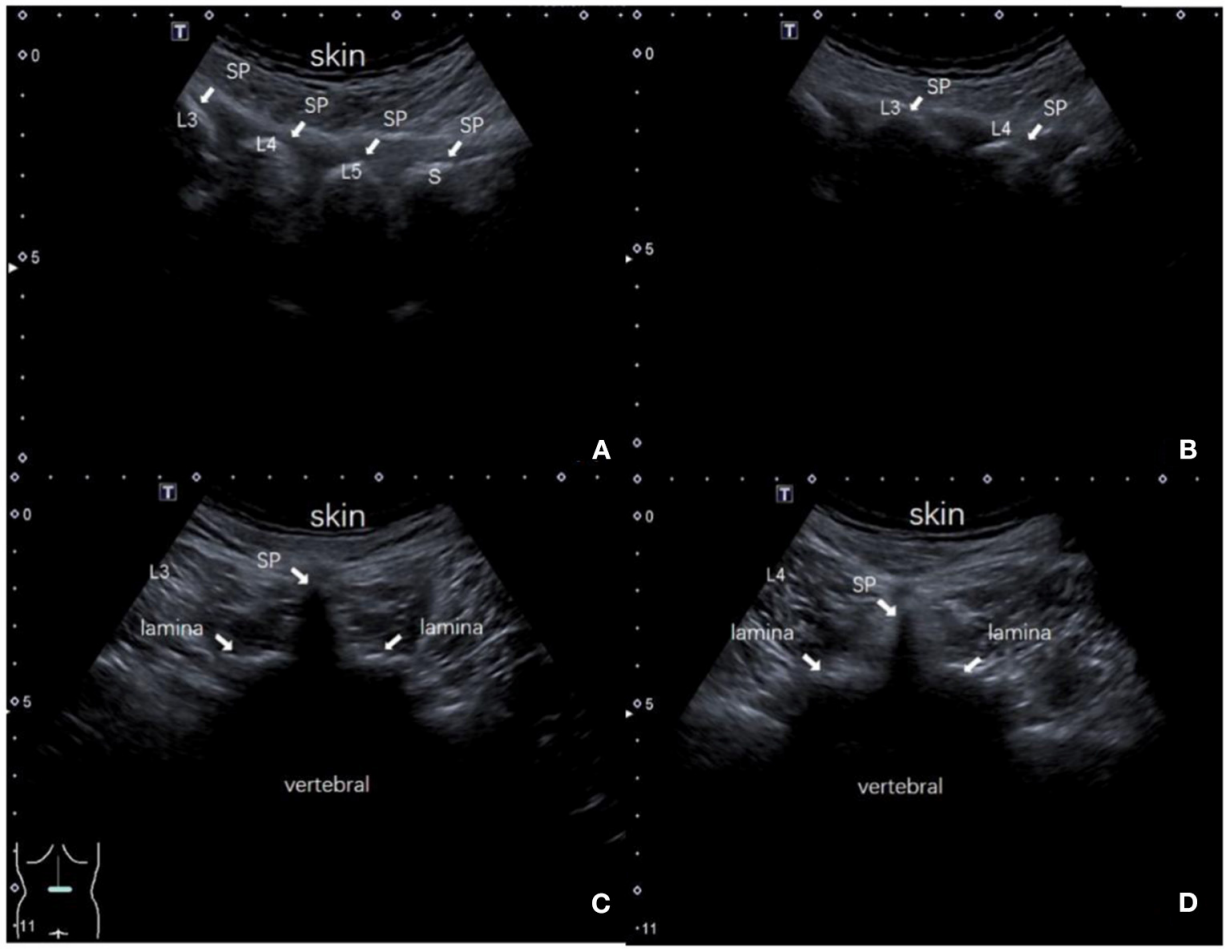
The interspinous space was located by ultrasound. **(A)** The ultrasound probe identified each spinous process (L3, L4, L5, and S1) on the longitudinal plane, white arrow; **(B)** The target interspinous space (L3–L4), white arrow; **(C)** Identify the midline spinous process of the upper vertebral body (L3); **(D)** Identify the midline spinous process of the lower vertebral body (L4). SP: spinous process.

In the landmark-guided group, each spinous process and interspinous space were marked on the body surface by Tuffier's line. Locate at the target interspinous space of L3-4 or L4-5, connect each spinous process as the midline, and the needle insertion point was 0.5–1 cm to the left of the midline (Figure 2A); in the ultrasound-guided group, the ultrasound probe identifies each spinous process on the longitudinal plane (Figure 3A), clearly mark the midline (Figure 2B) and puncture point (Figure 2C). Once the midline was determined and the target interspinous space was found (Figure 3B), the probe was rotated to cross section to identify the midline spinous processes of the upper and lower vertebrae (Figures 3C,D). When the probe was moved caudally or cephalically in parallel, avoiding the spinous process, the interspinous space can be scanned. The midpoint between the spinous processes was marked as the interspinous space (transverse line), again confirmed by the absence of hyperechoic spinous processes and sound shadows. Deeper structures, such as the ligamentum flavum and dural sac, do not need to be visualized. The puncture point was to the left of the midline beside the intersection of the midline and the transverse line (Figure 2B).

### Intraprocedural and Postprocedural Conditions

Compared with group A, group B had less lumbar puncture time, lower puncture attempts, and a higher first puncture success rate (*P* <0.05). In group B, the number of bloody CSF was less (*P* <0.05), postprocedural low back pain was less (*P* <0.05), intraprocedural sciatic nerve irritation and postprocedural paresthesia were less, but the difference was not statistically significant (*P* > 0.05) (Table 2).

**Table 2:** The comparison of conditions preprocedural and postprocedural in A and B group.

**Group**	**A**	**B**	***p***
Lumbar puncture time (min)	18.43 ± 1.06	7.53 ± 0.95^*^	0.000
Puncture attempts (*n*)	2.67 ± 1.49	1.13 ± 0.35^*^	0.000
First puncture success rate (*n*)	15/30	26/30^*^	0.005
Total puncture success rate (*n*)	27/30	30/30	0.237
The number of bloody cerebrospinal fluid (*n*)	9/30	2/30^*^	0.042
Intraprocedural sciatic nerve irritation (*n*)	5/30	1/30	0.195
Post-procedural low back pain (*n*)	8/30	1/30^*^	0.026
Post-procedural paresthesia (*n*)	2/30	0/30	0.492

* *Compared with A group, P <0.05*.

### VAS Pain Score

Compared with group A, the postprocedural VAS in group B was lower, and the difference was statistically significant (*P* <0.05) (Table 3).

**Table 3:** The comparison of VAS preprocedural and postprocedural in A and B group.

**Group**	***n***	**Preprocedural**	**Post-procedural**
A	30	4.03 ± 1.10	5.77 ± 0.94#
B	30	4.07 ±0.98	4.16 ± 0.91^*^

*#Compared with Preprocedural, P <0.05*;

* *Compared with A group, P <0.05*.

### Total Satisfaction

The total satisfaction of group A and group B was 60.0 and 86.7%, respectively. The total satisfaction of group B was higher than that of group A (*P* <0.05) (Table 4).

**Table 4:** The comparison of satisfaction in A and B group.

**Group**	***n***	**Very satisfied**	**Satisfied**	**Dissatisfied**	**Total satisfaction (%)**
A	30	11	7	12	60
B	30	20	6	4	86.7^*^

* *Compared with A group, P <0.05*.

## Discussion

Lumbar puncture for CSF is widely used in clinical diagnosis and treatment. It is very important in the diagnosis of infection, tumor, inflammatory disease, and hemorrhage (9–12). With the advancement of molecular technology, CSF has broadened its indications. It can be used for the determination of neuronal autoantibodies in autoimmune encephalitis, and early detection of neurodegenerative diseases (such as Alzheimer's disease), etc (13). Lumbar puncture is one of the important methods for obtaining CSF in the emergency department. The traditional method is to locate by palpating the anatomical markers. During the procedure, the sensory resistance disappears and the cerebrospinal fluid flows out to indicate a successful puncture. It has been proven to have a high success rate, but in some cases (such as scoliosis, high BMI) there are difficulties (14–16). Repeated puncture can cause pain, thereby increasing traumatic LP, affecting the test results, and reducing satisfaction.

At present, the emergency medicine department has more and more obese patients. Obesity has a thicker subcutaneous fat layer, unclear bony marks, and traditional punctures cannot accurately locate; the formation of cerebrospinal fluid mainly comes from the choroid plexus. Obese patients have smaller subarachnoid space than normal weight. Therefore, it is difficult to perform lumbar puncture. Some clinical workers use C-arm or CT scan for locating to avoid ossified ligamentum flavum and find the appropriate needle path (17, 18). However, C-arm or CT scan has radiation, and the emergency patients have special characteristics. They are often in serious condition and have risks to transfer. Therefore, C-arm or CT scans can be used for emergency patients for lumbar puncture only when necessary. Bedside ultrasound is a non-invasive and harmless clinical examination method. It can guide invasive procedures, and if necessary can display the bony landmarks of the lumbar spine and the subarachnoid space in real-time. The lumbar puncture operation under ultrasound positioning significantly improves the puncture success rate, shortens the puncture time, and reduces the pain.

Ultrasound guidance has been used to a certain extent. Park SK et al. used ultrasound-assisted and marker-guided paramedian spinal anesthesia in the elderly. It was found that ultrasound-assisted paramedian spinal anesthesia reduced the number of needle insertions, pain, and discomfort in the process. Ultrasound-assisted was helpful for the elderly in spinal anesthesia (19); Then, they also used ultrasound-guided anesthesia in 44 patients with abnormal spinal anatomy. The study showed that the number of needle insertions in the ultrasound group was significantly lower than that in the landmark group, the first success rate was also higher than that of the landmark group, and the perioperative period pain score was lower. Therefore, the use of ultrasound guidance significantly reduces the difficulty of anesthesia for patients with abnormal spinal anatomy. It is recommended that ultrasound-guided spinal anesthesia can be used for such patients (20). Alon Abraham et al. found that the use of ultrasound for lumbar puncture can increase the success rate, reduce the number of punctures, and reduce pain (21). Shervin Farahmand et al. found that ultrasound can detect six major lumbar markers in overweight and obesity, including spinous process, ligamentum flavum, lamina, epidural space, subarachnoid space, and posterior longitudinal ligament. In this study, midline and paramidline approaches were used to compare the use of curved and linear transducers in patients with a body mass index (BMI) higher than 25 kg/m2. It was found that the paramedian approach and curved transducer were better than the midline approach and linear transducer (22). Therefore, we chose the paramedian approach and the curved transducer. The increase of BMI made palpation of markers more difficult, but it did not affect the ability of ultrasound to identify relevant markers. These results suggest that ultrasound-guided LP is more reliable than traditional palpation marker technology in obese patients, can effectively ensure the success rate of puncture and save puncture time.

This article conducted a lumbar puncture study on 60 obese patients. The results showed that the puncture time and puncture attempts of the ultrasound-guided group were significantly less than the landmark-guided group, suggesting that ultrasound guidance can reduce the lumbar puncture time and the puncture attempts in obese patients. Lumbar puncture often relies on the experience of the operator, using a puncture needle for exploration. If the puncture needle encounters bone, then withdraw the needle and adjust the direction of the puncture until the puncture is successful. Ultrasound-guided punctures can display the bony landmarks of the lumbar spine and the subarachnoid space. The operator can choose the best puncture path according to the ultrasound image to avoid bone. The application of ultrasound makes the puncture process more intuitive and facilitates a smooth puncture. All 30 patients in the observation group were successfully punctured, with first puncture success rate of 86.7%; 27 patients in the control group were successfully punctured, with first puncture success rate of 50%; the observation group's first puncture success rate was significantly higher than that of the control group (*P* <0.05), indicating that ultrasound guidance can improve the first puncture success rate of obese patients. Ultrasound guidance is helpful to find the puncture space, the best angle, and depth, and predict the puncture path. It has a clear guiding significance for obese patients with unclear anatomical landmarks. A variety of symptoms can occur after lumbar puncture, of which low back pain is the most common. Reducing the complications has always been a clinical problem to be solved urgently. The results of this study showed that the incidence of low back pain in the observation group was significantly lower than that in the control group (*P* <0.05); the difference in the incidence of paresthesia between the two groups was not statistically significant (*P* > 0.05), suggesting that ultrasound-guided lumbar puncture can reduce the incidence of low back pain, but no significant improvement in postoperative paresthesia symptoms. The puncture under ultrasound guidance can avoid accidental penetration of blood vessels and nerves, reduce tissue and ligament damage, and reduce the occurrence of low back pain. The results of this study can promote the application of ultrasound guidance LP in overweight and obese patients.

In conclusion, compared with the traditional method of lumbar puncture, ultrasound-guided lumbar puncture takes less time, the first puncture success rate is high, and there are fewer adverse reactions such as low back pain. The method is safe and effective. Therefore, ultrasound-guided lumbar puncture can be used for obese patients with difficulty in the lumbar puncture. Although there may be acquiescence bias or response bias in this study, it is worthy of clinical application and promotion.

## Data Availability Statement

The original contributions presented in the study are included in the article/supplementary material, further inquiries can be directed to the corresponding author/s.

## Ethics Statement

The study was approved by the Ethics Committee of Shengjing Hospital, China Medical University. The patients/participants provided their written informed consent to participate in this study. Written informed consent was obtained from the individual(s) for the publication of any potentially identifiable images or data included in this article.

## Author Contributions

LL designed and conducted the study, contributed to patient recruitment, data collection, data analysis, and prepared the manuscript. WCT collected the data. XC analyzed the data. All authors approved the final version of the manuscript.

## Conflict of Interest

The authors declare that the research was conducted in the absence of any commercial or financial relationships that could be construed as a potential conflict of interest.

## Publisher's Note

All claims expressed in this article are solely those of the authors and do not necessarily represent those of their affiliated organizations, or those of the publisher, the editors and the reviewers. Any product that may be evaluated in this article, or claim that may be made by its manufacturer, is not guaranteed or endorsed by the publisher.
